# Spatiotemporal analysis of historical records (2001–2012) on dengue fever in Vietnam and development of a statistical model for forecasting risk

**DOI:** 10.1371/journal.pone.0224353

**Published:** 2019-11-27

**Authors:** Bernard Bett, Delia Grace, Hu Suk Lee, Johanna Lindahl, Hung Nguyen-Viet, Pham-Duc Phuc, Nguyen Huu Quyen, Tran Anh Tu, Tran Dac Phu, Dang Quang Tan, Vu Sinh Nam

**Affiliations:** 1 International Livestock Research Institute, Nairobi, Kenya; 2 International Livestock Research Institute, Regional Office for East and Southeast Asia, Hanoi, Vietnam; 3 Uppsala University, Uppsala, Sweden; 4 Swedish University of Agricultural Sciences, Uppsala, Sweden; 5 Centre for Public Health and Ecosystem Research (CENPHER), Hanoi University of Public Health, Hanoi, Vietnam; 6 Vietnam Institute of Meteorology, Hydrology and Climate Change (IMHEN), Hanoi, Vietnam; 7 National Institute of Hygiene and Epidemiology, Hanoi, Vietnam; 8 General Department of Preventive Medicine, Ministry of Health, Hanoi, Vietnam; National Taiwan University, TAIWAN

## Abstract

**Background:**

Dengue fever is the most widespread infectious disease of humans transmitted by *Aedes* mosquitoes. It is the leading cause of hospitalization and death in children in the Southeast Asia and western Pacific regions. We analyzed surveillance records from health centers in Vietnam collected between 2001–2012 to determine seasonal trends, develop risk maps and an incidence forecasting model.

**Methods:**

The data were analyzed using a hierarchical spatial Bayesian model that approximates its posterior parameter distributions using the integrated Laplace approximation algorithm (INLA). Meteorological, altitude and land cover (LC) data were used as predictors. The data were grouped by province (n = 63) and month (n = 144) and divided into training (2001–2009) and validation (2010–2012) sets. Thirteen meteorological variables, 7 land cover data and altitude were considered as predictors. Only significant predictors were kept in the final multivariable model. Eleven dummy variables representing month were also fitted to account for seasonal effects. Spatial and temporal effects were accounted for using *Besag-York-Mollie* (BYM) and autoregressive (1) models. Their levels of significance were analyzed using deviance information criterion (DIC). The model was validated based on the Theil’s coefficient which compared predicted and observed incidence estimated using the validation data. Dengue incidence predictions for 2010–2012 were also used to generate risk maps.

**Results:**

The mean monthly dengue incidence during the period was 6.94 cases (SD 14.49) per 100,000 people. Analyses on the temporal trends of the disease showed regular seasonal epidemics that were interrupted every 3 years (specifically in July 2004, July 2007 and September 2010) by major fluctuations in incidence. Monthly mean minimum temperature, rainfall, area under urban settlement/build-up areas and altitude were significant in the final model. Minimum temperature and rainfall had non-linear effects and lagging them by two months provided a better fitting model compared to using unlagged variables. Forecasts for the validation period closely mirrored the observed data and accurately captured the troughs and peaks of dengue incidence trajectories. A favorable Theil’s coefficient of inequality of 0.22 was generated.

**Conclusions:**

The study identified temperature, rainfall, altitude and area under urban settlement as being significant predictors of dengue incidence. The statistical model fitted the data well based on Theil’s coefficient of inequality, and risk maps generated from its predictions identified most of the high-risk provinces throughout the country.

## Introduction

Dengue fever (dengue) is a major infectious disease of humans in the tropics and sub-tropics caused by dengue virus (DENV) and transmitted by *Aedes* mosquitoes. The virus has a single positive-stranded RNA genome and is classified into the family *Flaviviridae* and genus *Flavivirus*. There are 4 antigenetically-related DENV (DENV 1–4) each capable of causing the disease [1]. DENV infections cause various symptoms ranging from asymptomatic or uncomplicated fevers to more severe illnesses especially following secondary infections with heterotypic DENV [2]. A haemorrhagic form of the disease has also been reported. The main vector, *Aedes aegypti*, a predominantly urban mosquito, breeds mainly in water-filled reservoirs, often artificial containers, in human settlements [3]. Other outdoor mosquitoes in the same sub-genus, including *A*. *albopictus* and *A*. *polynesiensis*, are capable of transmitting the virus [4]. Humans are the primary vertebrate host of DENV but in Africa and Asia, an enzootic transmission cycle exists that involves non-human primates [4]. There is uncertainty on the degree of immunity which naturally acquired mosquito-borne infections confer in vertebrate hosts.

There has been an unprecedented expansion of the geographical range of the disease globally since the 1950s [1]. Until 1970, severe dengue epidemics were reported in less than 10 countries [1][4]. Currently, it is thought that the disease is endemic in more than 100 countries, with a third of the world population living in areas with heightened dengue risk [5]. The spatial expansion of the disease mostly occurred between 1980 and 2010 [1]. Factors that could be attributed to this trend include urbanization, tourism and migration, and climate change [6]. Migration and tourism are believed to play a critical role in the spread of the disease if infected travellers successfully transfer the virus to new environments. Environmental factors such as human settlements, presence of water bodies, mixed agriculture, open land and neglected grasslands [7], determine DENV persistence, while factors which influence vectorial competence of DENV vectors (such as emergence rate, gonotrophic cycle, survival rate, etc.) have important effects on the incidence of the disease [8].

Dengue incidence has also increased in endemic countries over time [1]. A study conducted in the Southeast Asia show that between 1980 and 2010, dengue incidence increased by 6.7% in Thailand, 10.4% in Vietnam, 12.0% in Indonesia, 18.1% in Malaysia and 24.4% in Philippines [9]. In China, a total of 655,324 cases (6.6 cases in 100,000) and 610 (6.10E– 03) deaths were reported in the mainland, and in 2009–2014, 52,749 cases (0.53 cases in 100,000) and 6 (6.00E—05 in 100,000) deaths were reported in this region between 1978 to 2008 [10]. Risk factors associated with high incidence of the disease in Vietnam, Indonesia, Malaysia and Philippines include urban settlements with high population densities, poor drainage systems, inadequate waste disposal and extensive use of water storage containers that can be used by mosquitoes for breeding [9]. Furthermore, analyses conducted in Vietnam [11], India [12], Cambodia [13], and Nepal [5] show a positive correlation between dengue incidence and rainfall, temperature and humidity. In studies conducted in Vietnam [11] and India [12], stronger effects of rainfall and temperature were observed when these variables (rainfall and temperature) were lagged by two months.

Many statistical models have been used to analyse the effects of climate factors on dengue occurrence patterns. In Vietnam, for example, dengue patterns have been analysed using time series seasonal decomposition model (in four provinces with high incidence) [11], cluster analysis based on SatScan statistics (within the city of Hanoi) [14], and a Poisson or negative binomial regression model with lagged variables (also in Hanoi) [15][16] [17]. Similar approaches have been used in other countries; a generalised linear model has for instance been used to predict the disease in Yogyakarta [18] while a novel modelling approach that used fuzzy rule-based data mining technique was used in the Philippines (with a positive predictive value of over 70%) [19]. Wavelength coherent analyses driven by ENSO incidences has also been used to study multi-annual cyclical patterns that characterise the disease occurrence in the Southeast Asia [20]. Most of these approaches have, however, used data from few independent geographical locations or cities and focussed more on temporal dynamics. Spatiotemporal interactions have, therefore, not been fully examined yet it is known that dengue cases cluster in space and time [14][21] due to variations in (i) socio-economic development, including urbanization trends, (ii) levels of awareness on the disease, (iii) access to public health services, and (ii) frequency of local movements between contiguous areas.

This study used a spatiotemporal hierarchical Bayesian model to analyse surveillance data from the National Dengue Control Programme (NDCP) in Vietnam. Bayesian models were preferred because they provide a rigorous framework that can account for spatial-temporal autocorrelation and are increasingly being used for disease mapping [22]. The study aimed to develop a statistical model that can be used for developing dengue risk maps and forecasting dengue incidence at monthly intervals while at the same time accounting for spatiotemporal autocorrelations. Risk maps and incidence forecasting tools would enable policy makers to deploy risk-based interventions. The availability of 12 years surveillance records from a dengue dedicated project, meteorological data, and high-resolution geographical data provided a unique opportunity for the development of these tools.

## Materials and methods

### Vietnam

Vietnam is located on the eastern Indochinese peninsula and has a long, narrow spatial domain with an estimated population of 93 million in 2015 [23]. It has one of the highest population densities in the world with about 263 people/km^2^. Highest densities are found in agricultural areas—such as the Red River Delta, the Southeastern and the Mekong River Delta–as well as in the largest cities, Hanoi and Ho Chi Minh. Conversely, the northern part of the county is sparsely populated largely because of its high altitude and expansive forests especially in the northwestern region. Climate has a strong latitudinal gradient given the north-south elongation of the country that makes it straddle many climatic zones [24]. The central and southern regions experience humid conditions throughout the year which favours many infectious agents and vectors such as mosquitoes. Dengue virus has been isolated from various mosquito species in the country including *Aedes albopictus*, *Ae*. *aegypti and Culex vishnui* [25]. *Ae*. *aegypti* and *Ae*. *albopictus* are classical vectors of DENV but for the first time, Lien et al. [25] detected positive infection in *C*. *vishnui from* southern Vietnam. However, the role of *C*. *vishnui* in the virus transmission has not been described.

### Data

The study used dengue surveillance data that were collected by the NDCP program over a 20-year period between 1994–2013, and published annually by the Ministry of Health in annual record booklets [26]. The NDCP program was set up in 1999 to coordinate dengue surveillance and control. Detection and reporting of dengue followed the Ministry of Health Guidelines 1999 [27]. A case definition recommended by the World Health Organization for provisional diagnosis was used to detect clinical cases. The case definition comprised acute febrile illness of ≥38°C lasting 2–7 days with at least two of the main symptoms including severe headache, retro-orbital pain, nausea, vomiting, myalgia, arthralgia, haemorrhagic manifestations, and leukopenia [1][28]. Before 2002, a few cases were confirmed using serological tests, but from 2002 onwards, the surveillance system collated cases confirmed using anti-dengue virus IgM Elisa test [16]. Cases detected in the clinics and laboratories had to be reported to the province/city Preventive Medicine department within 24 hours and reports on the trends observed were issued at monthly intervals. A dengue outbreak was officially declared when a locality (a group/street/hamlet/sub-hamlet, inhabitant group or equivalent) reported clinical cases fitting the case definition given above, or when a laboratory confirmed case, together with finding the presence of mosquito vector/or mosquito vector larvae within 200m radius around a patient’s house. The study only accessed records aggregated at the province level–these data therefore had no personal information. Estimates of human population by province-month were derived via a reverse calculation described by Lee [29] and used as the denominator for estimating incidence. The reverse estimation procedure used published dengue cases and dengue incidence in 100,000.

Data required for this analysis were obtained from various on-line databases. Monthly meteorological data were obtained from the Institute of Meteorology and Hydrology and Climate Change, Hanoi. This dataset had 13 variables including total monthly rainfall (mm), highest rainfall (mm) in a month determined from daily records, total evaporation (mm), average temperature (°C), average minimum and maximum temperature (°C), absolute minimum and maximum temperature (°C), relative humidity (%), absolute minimum humidity (%), total duration of sunshine (hours), wind velocity (m/s) and atmospheric pressure (kPa).

Land cover data were obtained from NASA’s Moderate Resolution Imaging Spectroradiometer (MODIS) website. These data (referenced as MCD12Q1 product) has a spatial resolution of about 10 x 10km and has seven main LC types including forests, woodlands, grasses/cereals, shrublands, cropland/mosaics, wetlands and unvegetated areas [30]. In these data, a given LC type (savannah, forest, urban, crops, and wetland) ought to cover more than 50% of a pixel for that LC type pixel to be ascribed to the pixel, otherwise, the pixel would have a zero value for that LC type.

We extracted all these LC types except unvegetated areas using the province shapefile and determined the area of a province covered by each of the LC type. The MCD12Q1 data are stored in degree unit with a resolution of approximately 0.08° and were available in annual intervals; we downloaded those relevant for the target period 2001–2012. The data were downloaded in TIFF format, clipped using the Vietnam shapefile and converted into a vector layer in ArcGIS using the Asia South Albers Equal Area Conic. A new field for recording area (in square Kilometers) was created in its data attribute table. The geometry calculation table was then used to estimate the area of each LC cover type and expressed as a percentage of the total area of a province.

An ascii file on elevation in meters with a resolution of about 10 x 10km was downloaded from the Food and Agriculture Organization of the United Nation’s soil data portal [31]. These were resampled using the province shapefile based on bilinear interpolation method for altitude and nearest neighbour method for LC in order to harmonize their dimensions. These data were then merged with the dengue fever records, meteorological variables and human population using the province ID as the primary key. Maps of all the environmental data used are given in S1 Fig.

### Descriptive analyses

#### Dengue incidence, seasonal and interannual trends

Crude dengue incidence was derived as the proportion of dengue cases reported of the estimated human population per month and multiplied by 100,000 to obtain the number of new cases per 100,000 people. All the crude and adjusted incidence rates reported here represent the number of new dengue cases in 100,000 people. Its distribution by province, month and LC variables was analyzed using line graphs and thematic maps. The first set of analyses generated crude incidence using all the data that were available (2001–2012). This estimate was stratified by province and mapped to determine the observed spatial distribution of the disease in the country. Similarly, mean monthly rainfall, minimum and maximum temperature were estimated by month and plotted with the monthly dengue incidence to determine their relative distribution patterns (described further in the next section). The distribution of interannual dengue incidence was also determined and plotted.

#### Distribution of dengue incidence, land cover types and altitude

The distribution of dengue incidence across various levels of each LC type and altitude was investigated by converting these geographical variables into categorical variables and subsequently stratifying monthly mean dengue incidence using the new indicator variables created. All the variables except wetlands and urban settlements were classified into three levels, with their lower and upper cut points being 30^th^ and 70^th^ percentiles, respectively (for a balanced distribution of data across the levels derived). Area under crop cultivation was classified into levels 0 − <24%, ≥24- <80% and ≥80%; area under savannah grassland 0 − <1%, ≥1- <20% and ≥20%; area under forests 0 − <2%, ≥2- <50% and ≥50%; and altitude 0 − <11m, ≥11- <285m and ≥285m. Area under wetlands and that for urban/built-up areas were categorized into two levels 0 and >0% because their values were mostly skewed to the right with a high density of zero values. All the descriptive plots were developed using the ggplot2 package in R [32].

#### Principal component analysis of the meteorological data

**Fig 1:**
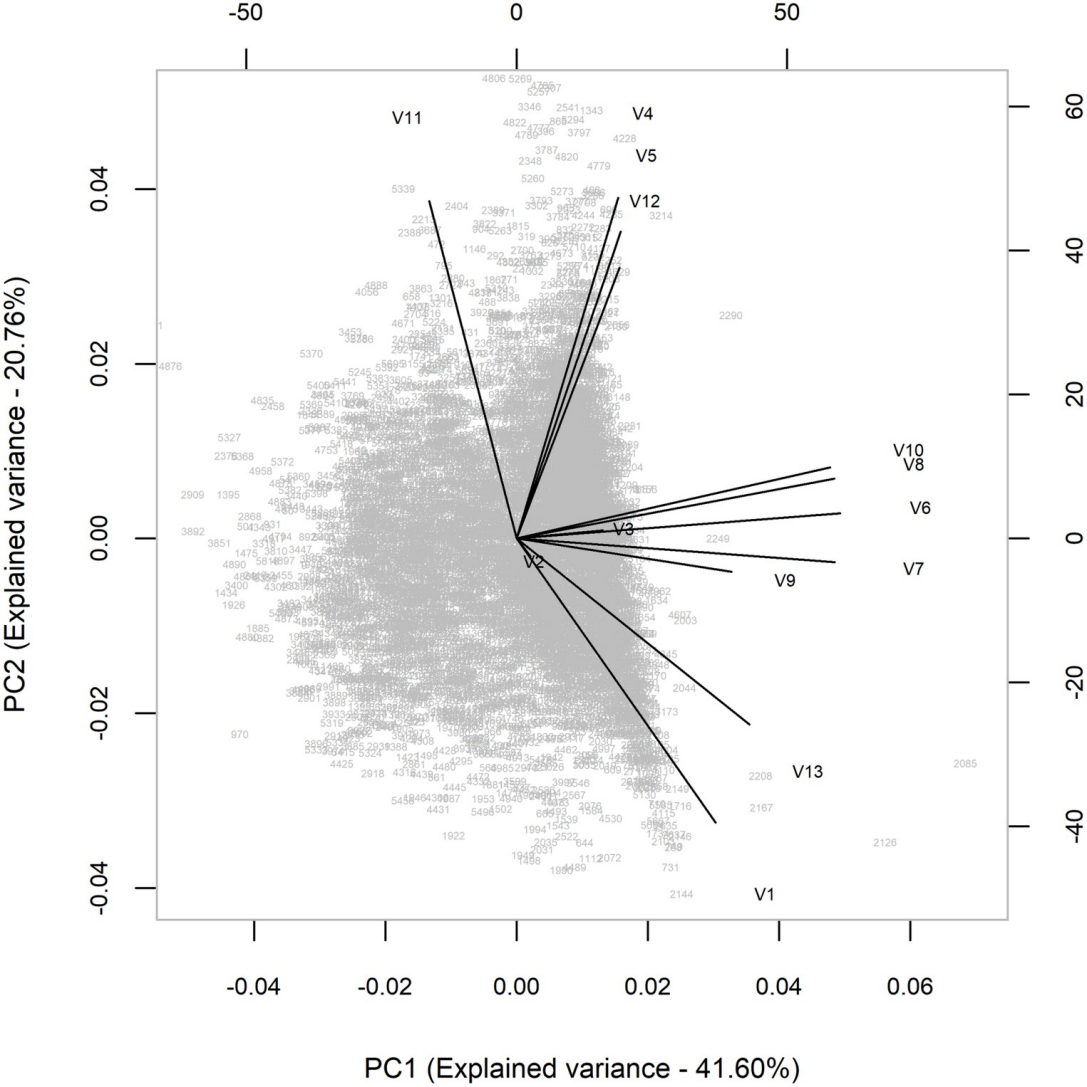
Biplot graph used to identify key predictors of dengue incidence in Vietnam among 13 meteorological variables (2001–2009).

We used principal component analysis to identify key meteorological variables for multivariable analysis. The selected variables had to cover at least 80% of the variance cumulatively to be considered as being good predictors. Each variable was normalized (i.e., their means set to zero) to standardize their scales. A standard biplot graph involving principal components 1 and 2 was then generated to identify the most orthogonal variables. The graph clustered variables into distinct categories of highly correlated predictors. Within each cluster, those variables that were far removed from the center of the graph were considered to represent most of the variation of the outcome than the others in that cluster. Rainfall, minimum temperature and evaporation met this selection criterion (Fig 1) and were also ranked highest in the PCA model that was generated alongside the biplot graph.

### Statistical modelling and prediction

#### Modelling approach

We analyzed the data using a hierarchical spatial Bayesian model which approximates its posterior parameters using integrated nested Laplace approximation methods (INLA) proposed by Rue, Martino, and Chopin [33] and implemented using the RINLA package in R version 3.4.1. The same model has been used to model dengue incidence in Bucaramanga, Colombia by Adin et al. [34]. It is particularly suited for analyzing multivariable, geographically referenced and (or) temporally correlated data. They are also suited for disease mapping since they obtain information from neighboring locations and generate predictions with reduced uncertainty. A description of these models is provided by Banerjee et al. [35]. In this analysis, the outcome of interest, as described above, was dengue incidence by province and month. This was analyzed as a conditionally independent Poisson random variable with the expected spatial and temporal autocorrelations accounted for using conditional autoregressive (CAR) and autoregressive (AR [1]) models, respectively. The specifications of the model was:

$$\eta_{i}=\mu +\sum_{j=1}^{n\beta }\beta_{j}x_{ji}+\sum_{k=1}^{nf}f^{k}+u_{i}$$ (1)

where,

*η*_*i*_ the linear predictor, *μ* the baseline mean, $\sum_{j=1}^{n\beta }\beta_{j}x_{ji}$ linear effects of *x*_*m*_ covariates with *β*_*m*_ coefficients; $\sum_{k=1}^{nf}f^{k}$ structured latent variables, which in our case was the spatial and temporal effects, and *μ*_*i*_ is unstructured random effect (residual noise) [36]. Modelling was implemented in two steps starting with model development using data for the period 2001–2009, followed by model validation using the data for the period 2010–2012. A validated model was then used to generate predictions for risk mapping. These analyses are described below.

#### 1. Model development

Univariable and multivariable analyses were implemented successively to generate a dengue incidence forecasting model. These analyses led to the identification of significant predictors, latent variables, and dummy variables for accounting for seasonal effects. Predictor variables included in these analyses were meteorological variables from the principal component analysis, all the environmental variables including altitude, and the spatial and temporal effects. For both univariable and multivariable models, a variable was considered as being significant if its 95^th^ credible interval (representing 2.5% and 97.5% quantiles) excluded zero. These limits were chosen to obtain a range that had the highest density of the estimated parameter values around the median. Lagged variables of each of the meteorological variables analyzed were also tested in turns. Up to three lags were considered for each variable and a form that returned the least deviance information criterion (DIC) estimate was considered as being suitable.

A combination of backward and forward variable selection process was employed to build a parsimonious multivariable model. For continuous variables, linearity assumption was evaluated by fitting respective quadratic terms. Eleven dummy variables representing month were also fitted in the model to account for the seasonal effects. The spatial effect was accounted for using the CAR model; this captures the neighborhood structure of the assessment units, which in this case were provinces. Alternative CAR models with varying correlation matrix specifications for the implied spatial design have been developed and reviewed extensively in literature. Three CAR models including intrinsic conditional autoregressive model, proper CAR (or Cressie model), and convolution model are available in RINLA as *besag*, *besagproper* and *Besag-York-Mollie* (BYM), respectively [37]. We used the BYM model which combines the CAR model and unstructured random effect to account for independent province-specific noise.

We used the province shapefile to specify the BYM adjacency matrix. Each data point, *y*_i_, was deemed to be based in a spatial region, *s*, which was part of a population of sites, *s* ∈ *S* = (0, ⋯·, *S* − 1). Two sites that shared a common provincial border were therefore considered to be neighbors. The spatial effect: *f*_*s*_(*s*_*i*_), i.e., *f*_*s*_ = (*f*(0), *f*(1), ⋯, *f*(*S* − 1)) was modelled as an intrinsic Gaussian Markov Random Field (GMRF), which is considered as an alternative but deterministic model for Markov Chain Monte Carlo (MCMC) model [36].

Similarly, autoregressive (AR [1]) model was used to account for temporal autocorrelation. Each province had 108 time units (between January 2001 to December 2009) that were used in the AR [1] model. The AR model assumed that dengue incidence in the current month was correlated with the that of the previous month.

#### 2. Model validation

The final model fitted was used to generate predictions for 2010–2012 period. Input data relevant for the period were offered to the model. Observed and predicted mean monthly incidence, together with its 2.5% and 97.5% quantile values were computed and plotted to determine their relative trends. The accuracy of the forecast was evaluated using Theil’s coefficient of inequality that has been used widely for model validation; it is described in detail by Bliemel and MacKay [38]. It is a coefficient with values ranging from 0, signifying a perfect forecast, to 1 representing maximum inequality. The formula used was:

$$U=\frac{{\left[\frac{1}{n}\sum_{i=1}^{n}{\left(A_{i}-P_{i}\right)}^{2}\right]}^{1/2}}{{\left[\frac{1}{n}\sum_{i=1}^{n}A_{i}^{2}\right]}^{1/2}+{\left[\frac{1}{n}\sum_{i=1}^{n}P_{i}^{2}\right]}^{1/2}}$$ (2)

where:

*U*–Coefficient of inequality

*n*–number of records

*A*_i_−Observations which in this case are incidence records for 2011–2012

*P*_i_- Predicted incidence data.

In the course of validation, four alternative spatio-temporal interactions described by Ugarte et al. [39] were tested in turn in a bid to improve the Theil’s coefficient and reduce uncertainty of the prediction.

#### 3. Risk mapping

A validated model was used to generate predicted dengue incidence.

## Results

### Mean dengue incidence

**Fig 2:**
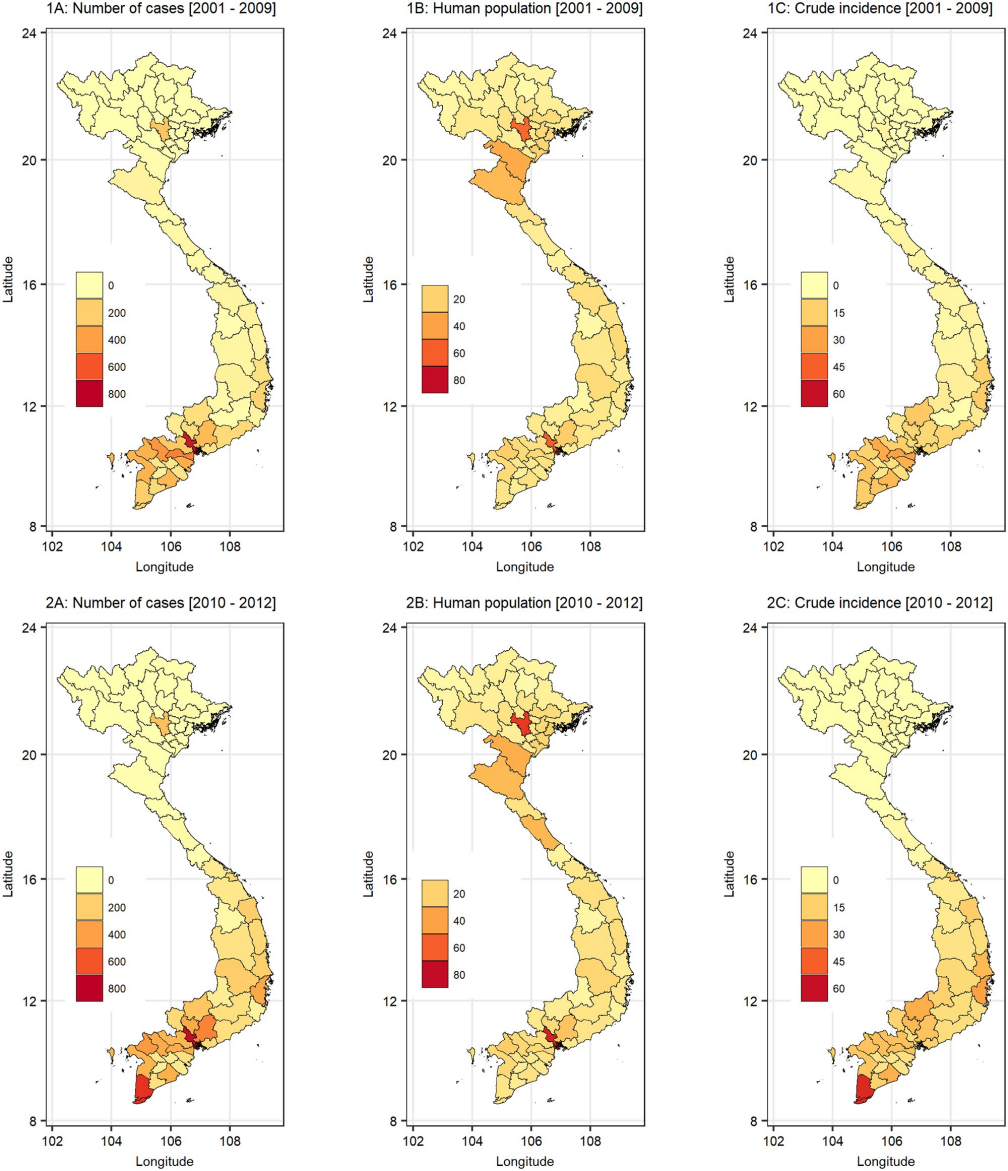
The distribution of crude number of dengue cases (1A), estimated human population (1B) and crude incidence (1C) by province in Vietnam in 2001–2009. The lower panel gives crude number of cases (2A), estimated human population (2B) and crude incidence (2C) by province in 2010–2012. The observed dengue incidence given in 2C was used to validate the forecasting model developed in this study.

The mean monthly dengue incidence averaged 6.94 cases (standard deviation 14.49) per 100,000 people over the period (2001–2010). Over the wet period (May-November with a mean monthly rainfall >40mm), the mean dengue incidence was 10.31 cases per 100,000 (SD 18.51). Provinces in the southern parts of the country had higher reported incidences compared to those from central and northern regions. Hanoi city also reported appreciably higher number of cases compared to the neighboring areas (Fig 2).

**Fig 3:**
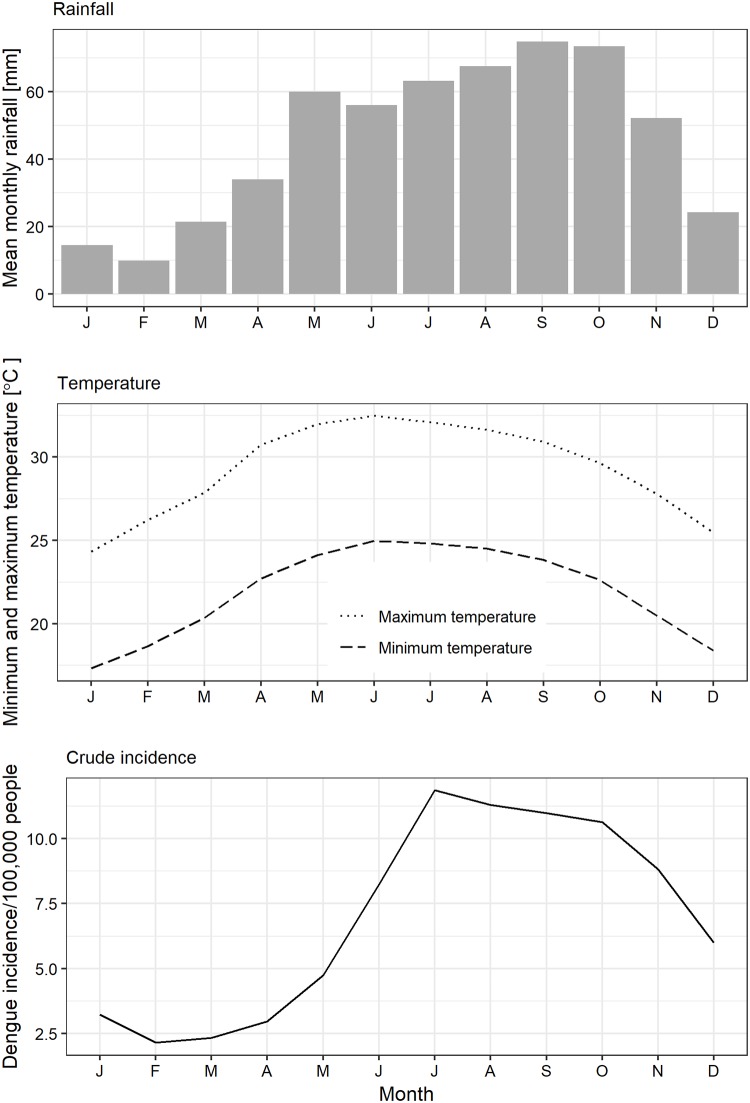
Observed seasonal trends in rainfall, minimum and maximum temperatures, and dengue incidence in Vietnam in 2001–2009.

Dengue incidence had a strong seasonal pattern which often peaked between July–October (Fig 3). This peak closely followed those of maximum rainfall, minimum and maximum temperatures.

### Distribution of dengue by levels of environmental factors

**Fig 4:**
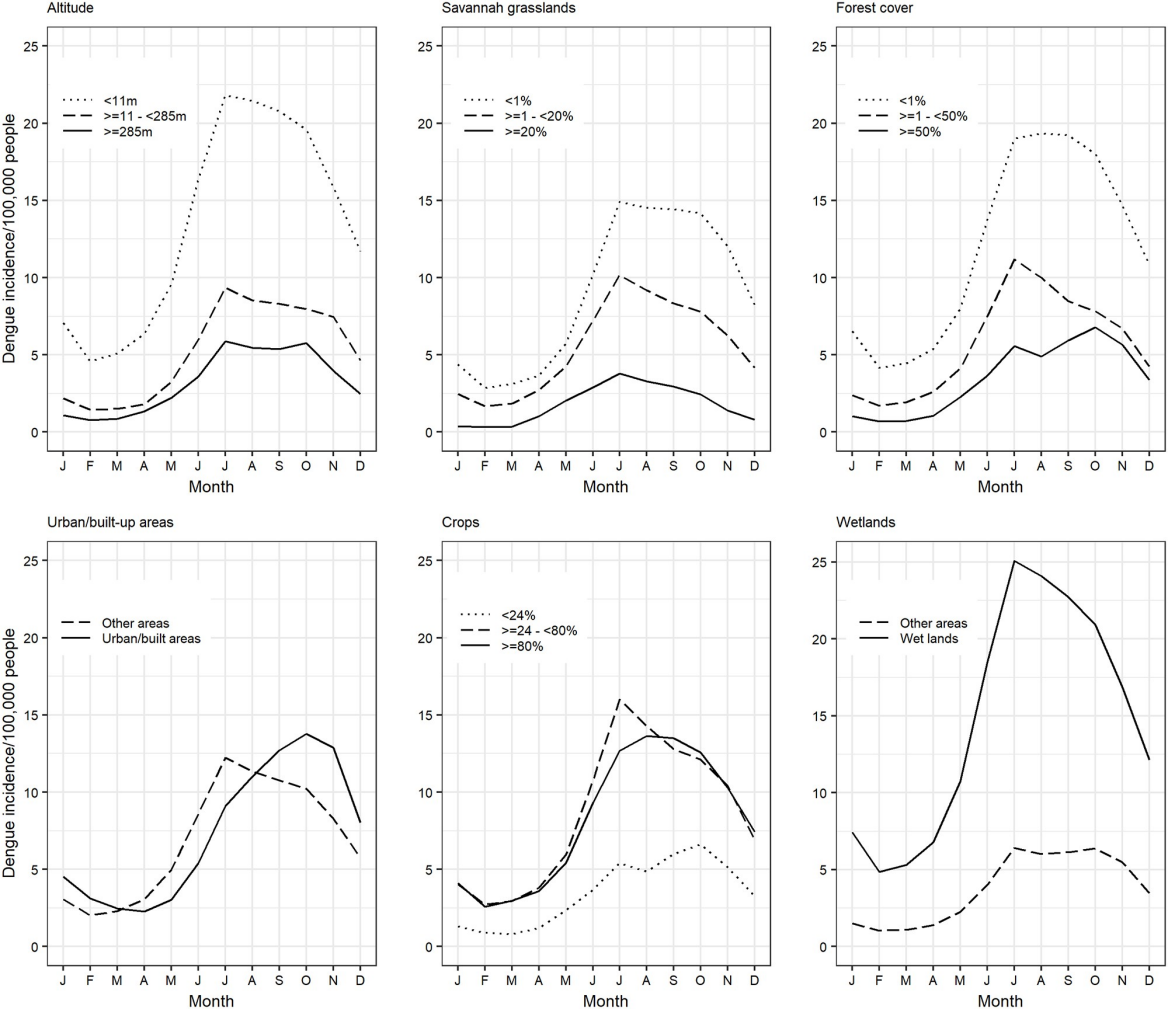
Trends in dengue incidence at various levels of environmental factors used in the study including altitude, area under savannah grassland, forests, urban development, crop farming and wetlands.

Relative trends between dengue incidence and environmental variables including LC and altitude are shown in Fig 4. Dengue incidence declined with an increase in altitude, area under savannah grassland and forests. Conversely, dengue incidence was higher in provinces with wetlands compared to those without. It also increased with area under crop farming. Provinces with urban/build-up areas generally had a slightly higher but delayed amplification of the incidence compared to those without.

### Interannual incidence

**Fig 5:**
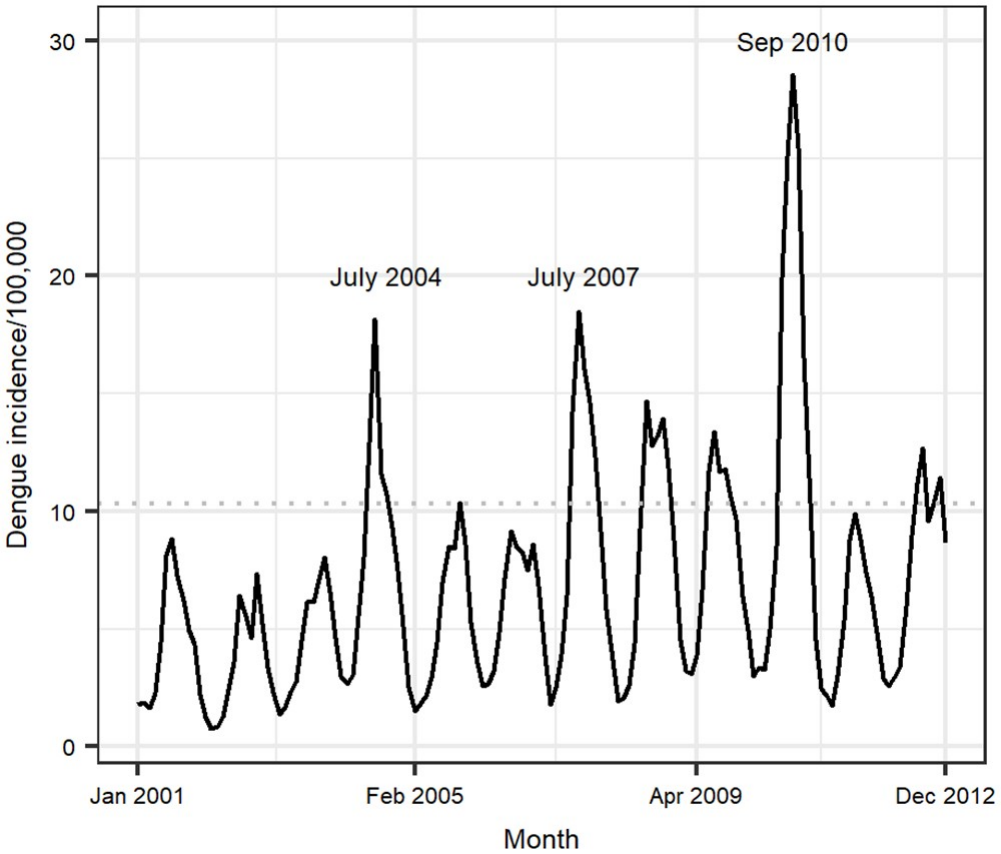
Inter-annual trends in dengue incidence in Vietnam. Months that had higher than expected peaks in the disease incidence are indicated in text within the graph. The horizontal dotted line at an incidence of 10.31 a threshold that was used to demonstrate changes in the peak incidence of the disease before and after mid-2005.

An analysis of dengue incidence over the entire period (2001–2012) showed consistent peaks and troughs consistent with seasonal climate patterns (Fig 5). Except in July 2004, peak dengue incidences were always lower than the mean for the wet period (10.31) until about mid-2005 when these peaks exceeded the threshold. In the later years, higher peak incidences (>10.31) were observed. Major outbreaks were also observed about every 3 years; these occurred in July 2004, July 2007 and September 2010.

### Univariable and multivariable analyses

Univariable analyses identified all the variables used including rainfall, minimum temperature, duration of sunshine, evaporation as being significant predictors for dengue incidence (Table 1). Lagging temperature by two months and rainfall by one month provided better fitting models compared to using unlagged variables. However, to simplify the analysis, both minimum temperature and rainfall data were lagged for two months and used in multivariable analyses. On cartographic variables, only wetlands were not significant at this level of analysis.

**Table 1:** Posterior parameter distributions from univariable models used to screen predictor variables.

Variable	Fixed effects				Hyperparameters					
	Intercept		Predictor		IID		BYM model		AR[1] model	
	Mean	Quantile range	Mean	Quantile range	Mean	Quantile range	Mean	Quantile range	Mean	Quantile range
Rainfall ^a^	-0.46	-1.13–0.17	0.02	0.19–0.02	1.03	0.54–1.76	0.72	0.25–1.66	1.91	0.83–3.32
Temperature ^a^	-3,21	-1.13–0.17	0.12	0.12–0.13	1.09	0.60–1.80	0.95	0.34–2.21	0.89	0.80–0.95
Evaporation	-0.57	-1.25–0.08	0.002	0.001–0.002	1.03	0.55–1.76	0.74	0.26–1.71	1.78	0.78–3.08
Duration of sunshine	-0.52	-1.20–0.13	0.001	0.001–0.001	1.03	0.54–1.76	0.73	0.25–1.67	1.79	0.79–3.11
Altitude	0.25	-0.49–0.97	-0.003	-0.004–-0.002	1.02	0.62–1.57	1.52	0.50–3.49	1.84	0.81–3.20
Wetlands	-0.42	-1.10–0.22	-0.001	-0.001–0.00	1.03	0.54–1.76	0.73	0.26–1.69	1.84	0.80–3.18
Shrubland	-0.42	-1.10–0.22	-0.02	-0.02–-0.01	1.03	0.54–1.76	0.73	0.26–1.69	1.84	0.80–3.19
Cropland	-0.17	-0.83–0.46	-0.005	-0.005–-0.004	1.05	0.53–1.87	0.60	0.21–1.32	1.88	0.84–3.27
Forests	-0.83	-1.50–-0.18	0.01	0.01–0.02	0.99	0.50–1.78	0.53	0.19–1.15	1.92	0.87–3.38
Urban settlements	-0.45	-1.12–0.19	0.07	0.07–0.08	1.05	0.56–1.79	0.74	0.26–1.69	1.86	0.83–3.24

^a^ Variable lagged by two months

Of all the predictor variables used in the multivariable analysis, total monthly rainfall, minimum temperature, and area under urban settlement/build-up areas, altitude, together with the spatial (CAR) and temporal (AR [1]) effects, were significant in the model (Table 2). Minimum temperature and rainfall (lagged by two months) did not meet the linearity assumption; each of these variables were therefore fitted as quadratic functions. The posterior parameter distributions for the main variables generated from the model (Table 2) suggest that minimum temperature and dengue incidence have an exponential relationship while that for rainfall and dengue incidence is a “concave-down”. This means that at lower levels, there was a positive correlation between rainfall and dengue incidence but at higher rainfall levels of about >500mm, rainfall had a negative correlation with the disease incidence. Areas with urban settlements had a higher risk of dengue (mean relative risk of 2.08) compared to rural/non-urban areas.

**Table 2:** Marginal posterior distributions of model parameters estimated from a parsimonious hierarchical Bayesian spatial model fitted to dengue fever data from Vietnam (2001–2010).

Variable	Levels	Mean	SD	Quantile	
				2.5%	97.5%
*Fixed effects*					
Intercept		-1.193	0.289	-1.771	-0.625
Minimum temperature^1^		-0.103	0.005	-0.112	-0.093
Rainfall^2^		0.082	0.003	0.076	0.087
Minimum temperature (squared)		0.006	0.000	0.005	0.006
Rainfall (squared)		-0.006	0.000	-0.006	-0.005
Altitude^3^		-0.186	0.065	-0.314	-0.056
Urban settlement	> 0%	0.597	0.012	0.573	0.620
	≤ 0%	1.000	-	-	-
*Model hyperparameters*:					
Spatial effect (iid)		1.189	0.292	0.712	1.856
Spatial effect (CAR)		1.636	0.848	0.533	3.767
Precision for time		5.325	1.969	2.112	9.642
Rho for time		0.913	0.032	0.842	0.967

^1.^ Minimum temperature lagged by two months

^2.^ Rainfall in mm divided by 100 to obtain appreciable parameter estimates

^3.^ Altitude in meters divided by 100

**Fig 6:**
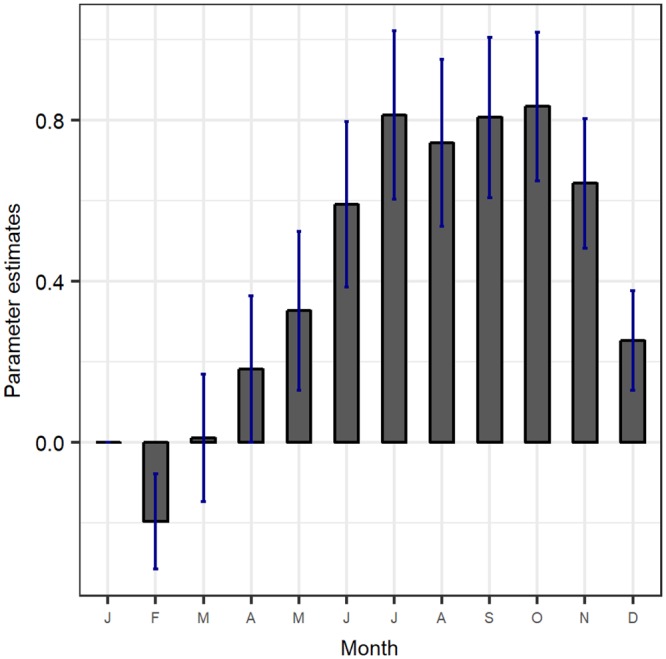
Posterior distributions of the dummy variables used in the model to account for seasonal effects.

The results given in Table 2 do not however include posterior distributions of the dummy variables used to account for the seasonal effects. These results are given in Fig 6. These estimates have a similar pattern as the crude incidence given in Fig 3.

### Validation of the model

**Fig 7:**
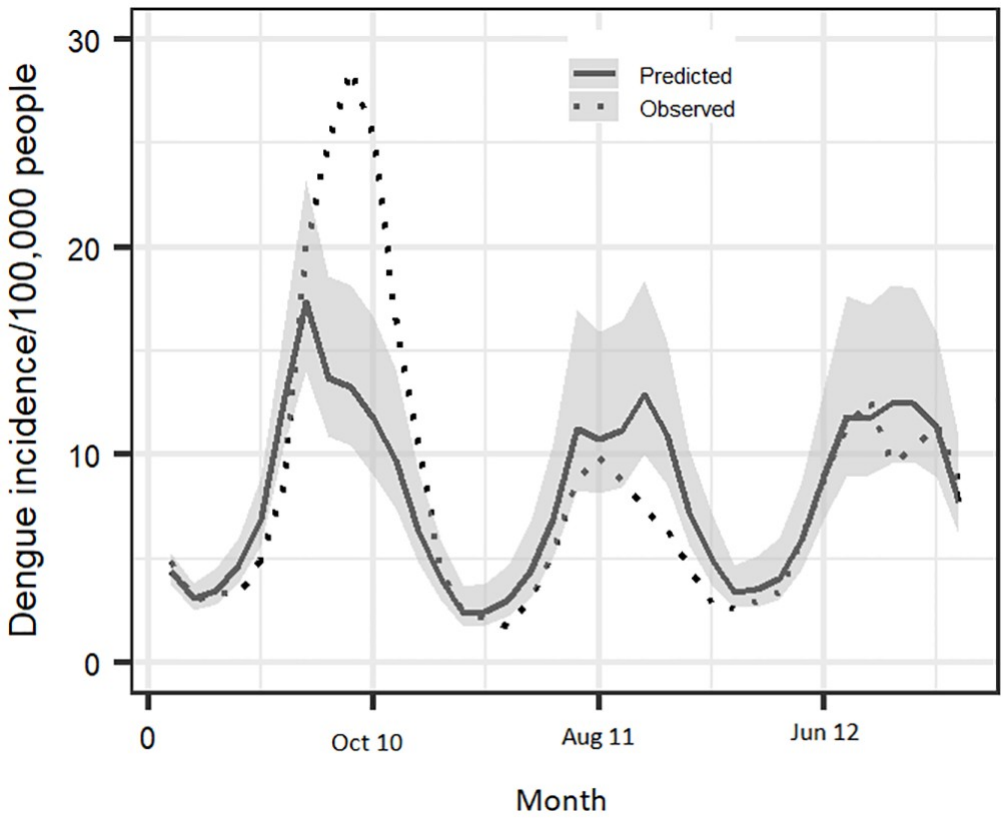
A comparison of the predicted and observed monthly mean incidence of dengue in Vietnam in 2010–2012 (36 months).

A comparison of the observed and predicted dengue incidences (2010–2012) (Fig 7) showed that the predicted incidence closely tracked the seasonal trends and identified peaks and troughs of the dengue occurrence patterns. However, during the September 2010 outbreaks, the predicted incidence was lower than the observed even though it was relatively higher than those of the other periods. The estimated Theil’s coefficient of inequality was 0.22.

### Risk mapping to determine dengue occurrence patterns

**Fig 8:**
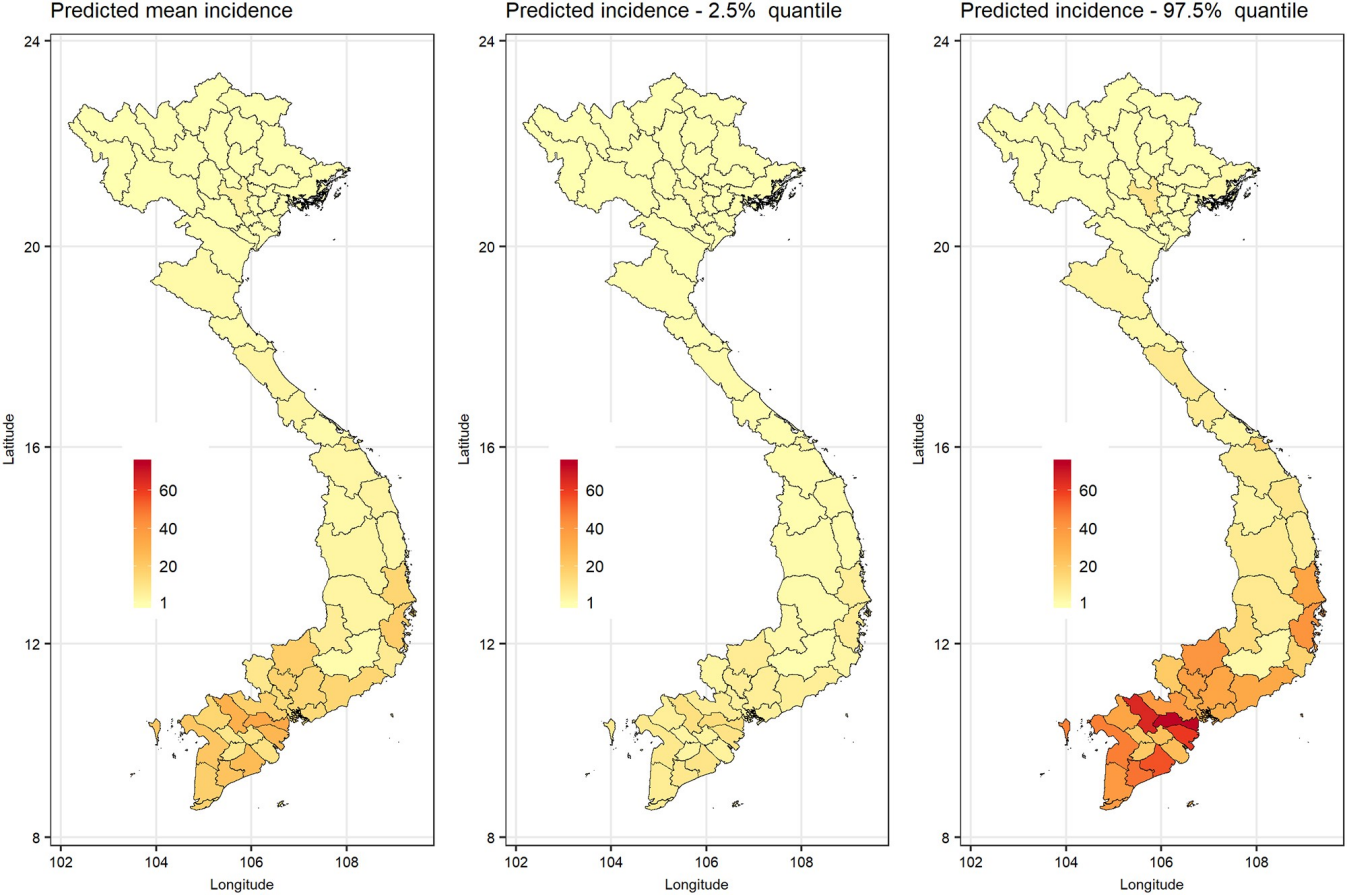
Predicted dengue mean incidence for 2010–2012, with 2.5% and 95% quantiles.

Fitted values representing the number of dengue cases per 100,000 people per month in 2010–2012 are displayed in Fig 8. Predicted dengue incidence matched the observed data (Fig 2) in many parts of the country. However, in the north, additional provinces that neighbor the Hanoi city were predicted to have higher dengue incidence that observed.

## Discussion

We analyzed dengue fever surveillance records collected between 2001–2012 in Vietnam and developed a statistical model that can be used for forecasting incidence. The disease has become a major threat to human health and wellbeing in the country [11] and with climate, the spatial range of dengue fever virus is likely to expand to areas that are currently considered as being relatively safe [40]. Early warning systems that utilize climate and land cover data, such as that described by Munasinghe et al. [41] are therefore required for risk prediction and to guide the deployment of interventions. The existing global dengue risk maps (e.g. Bhatt et al. [42]) cover multiple countries, including Vietnam, but they don’t delineate high risk areas within countries. Country-specific risk maps and forecasting models on dengue have been developed for many countries including Sri Lanka [41], Brazil [43], Argentina [44], Singapore [45] and in hyper-endemic cities of Colombia [34] and Venezuela [46]. Limited mapping of dengue has been done in Vietnam.

We used a hierarchical spatial Bayesian model with meteorological and geographical (altitude and LC) factors as predictors. These two groups of variables are seldom used together for dengue risk prediction. A systematic review of literature that involved 26 studies, for example, showed that only 7 used meteorological, climatological and/or remote sensing data [3] concurrently. Meteorological variables capture intra-annual, temporal dynamics of the disease (e.g. studies conducted by Lei et al. [47], while geographical variables determine its spatial distribution (e.g. studies by Ling [7]). In this study, 13 meteorological variables were available but only a few–i.e., minimum temperature, rainfall, evaporation—were selected as being important predictors through principal component analysis. Findings on the association between dengue incidence and meteorological variables (i.e., temperature and rainfall) are similar to those reported previously in Vietnam [11], India [12] and Cambodia [13].

Analyses of the temporal dynamics of the disease revealed regular seasonal epidemics that were interrupted by major fluctuations that occurred in 3-year cycles as reported previously in southern Vietnam [20]. Regular dengue epidemics have been attributed to extrinsic factors due to climate variability while the multi-annual fluctuations (observed at 3-year intervals in this case) are thought to be caused by antibody-dependent enhancement (ADE) of infection due to the introduction of new DENV into a sub-population or an area with cross-reactive antibodies from previous infections [48] [49]. We believe the ADE effects cause an increase in hospitalization rates and hence registration of increased dengue cases in the surveillance system. Infection dynamics associated with ADE effects are, however, beyond the scope of this paper. More work is needed though to identify how new DENV are introduced to new areas or sub-populations of exposed individuals.

We found non-linear associations between dengue incidence and both minimum temperature and total monthly rainfall. Controlling for the other variables considered, an increase in the minimum temperature within the limits observed in Vietnam (14 to 28.5°C) led to an exponential increase in dengue incidence. On the contrary, an increase in maximum rainfall per month (within the limits of 0–1,500 mm) was associated with a “concave-down” relationship with the outcome. That means from 0 to about 550 mm, rainfall was associated with an increase in dengue incidence but beyond this level, an increase in rainfall was associated with a decline in dengue incidence. Evidence from studies on malaria vectors show that high rainfall is known to cause flushing of mosquito breeding sites, therefore making them less suitable for breeding [50], and it is likely that the same effect applies to dengue vectors. Better results were obtained when both rainfall and minimum temperature were lagged by two months to account for the period required for the amplification of vector populations once appropriate breeding conditions are provided, as well as the completion of extrinsic incubation period of the virus.

Temperature has multiple pathways through which it can influence dengue risk. In general, an increase in temperature shortens the development intervals of immature stages of mosquitoes, increases the biting rates of adults and shortens the extrinsic incubation period of pathogens [51]. It is also expected to encourage the adoption of behavioral practices in humans that enhance human-vector contact such as staying outdoors for long periods or leaving their residences’ windows open while inside [47].

Rainfall, on the other hand, influence dengue occurrence by expanding the available breeding grounds. It fills containers and depressions where dengue vectors can breed from [15] and varies humidity levels. The ideal breeding habitats for dengue vectors (e.g. *Ae*. *aegypti*) include uncovered water containers, drainage systems, disposed plastic containers, or used tyres [1]. It is also expected that poor drainage canals in peri-urban areas may serve as reliable mosquito breeding grounds and floods that develop after rainfall act as additional breeding sites that amplify mosquito numbers.

Environmental variables determine relative distributions of pathogens, vectors and hosts in space, and changes in their structure would destabilize established relationships (within and between communities) leading to emergence of infectious diseases [52]. In this study, altitude and LC represented environmental variables used to determine the distribution of ecological niches for dengue. A number of studies have investigated the effects of altitude [53] [44] [54] and urban development [55] [56] on dengue incidence in various countries but not much has been done on the other LC types. Our analyses established a negative association between altitude and dengue risk. A survey conducted in Nepal along an altitudinal gradient showed that *Ae*. *aegypti* and *Ae*. *albopictus* could be found between 85–1,300m but rarely beyond 1,750 to 2,010m above sea level [57]. A similar observation has been made for a broad range of vector-borne diseases including Japanese encephalitis [58] and malaria [58], among others. Apart from influencing climate patterns, altitude modifies other factors that may influence mosquito survival such as vegetation cover, and availability/density of hosts.

Of all the LC types considered, only the urban/build-up areas was significant in the final model. Compared to urban/build-up areas, croplands and wetlands were not significant yet these LC types could support mosquito breeding, and have been associated with endemicity of other vector-borne diseases including malaria [59], schistosomiasis [60], Rift Valley fever [61], and West Nile fever [62]. However, *Ae*. *aegypti*, the main vector of dengue virus, is strongly anthropophilic and thrives well in domestic environments. Urban settlements/build-up areas are more suited for dengue endemicity but these mosquitoes can acclimatize to other external breeding environments [63]. Moreover, an increase in human population in peri-urban areas leads to expansion of build-up areas, crowding and damping and therefore the creation of more mosquito breeding sites [6]. This ultimately results in increased vector-host contact, and faster rates of transmission of the virus. The intense vector-host contact is critical for dengue transmission because the infectious period in humans, when uninfected mosquitoes can pick the virus, is only12 days and there are no other reservoir hosts that can sustain the transmission [64].

Latent variables representing space and time are often used to account for spatiotemporal autocorrelations [37]. Some of the spatial effects could be attributed to differences in ease of travel/movement that might allow importation of cases in some areas, topography of the spatial units which allow various degrees of water drainage, or socio-economic activities that influence exposure to the disease. The model estimated the spatial effect of a province by integrating the effects of all the neighboring provinces using the intrinsic autoregressive model (CAR). This ensured that reliable dengue risk maps could be generated. The AR [1] model used to account for temporal autocorrelation.

The model developed could provide a reliable forecast of dengue risk based on favorable Theil’s coefficient of inequality obtained at the validation step of the analysis. Absolute percentage error (MAPE) is another commonly used accuracy measure but this was not used in this study because it is thought to be unreliable especially when the observed data have low values. It produces large percentage errors when the observed data series is low and outliers may distort accuracy assessments [45]. The model reliably captured seasonal changes in dengue incidence, but it could not accurately match the peaks associated with the major epidemics that occurred in September 2010, which as stated above, was mainly driven by ADE effects. The model was still able to predict a higher incidence compared to that predicted for the rest of the periods indicating that to some degree, climate factors contribute to the amplification of the cyclic multi-annual dengue incidence during such periods in addition to ADE.

The predicted risk maps produced results that are similar to the dengue vulnerability maps that have been published by Fullerton et al. [65]. It reliably predicted high dengue incidence in most of the high-risk areas in the southern parts of the country and additional areas in the north, such as the Hanoi city, that is known to have high dengue incidence [11]). As identified in the dengue vulnerability maps published by Fullerton et al. [65], the model predicted a higher dengue incidence in a few more provinces in the north than observed. One of the possible reasons that can explain the mismatch is that people from affected areas might have sought medical services in Hanoi city. It has also been observed that data from regional surveillance systems may not be recorded and reported uniformly between areas. Cohort studies conducted by Wichman et al. 201 [66] in Thailand and Cambodia, indicated that dengue incidence was under recognized by 8–9 times and area-specific multiplication factors were required to amplify the number of cases observed to obtain reliable measures of burden. Regions with discordant predicted and observed incidence trends can be targeted for cohort or capture recapture studies to obtain primary data on incidence to inform the refinement of existing surveillance systems and risk mapping.

The study, however, had some limitations. One, some of the data used (especially those collected in 2001) were clinical records that had not been subjected to laboratory screening. Most of the data collected after 2002 were however screened using anti-dengue virus IgM ELISA as mentioned earlier. Despite this limitation, dengue surveillance data are often used for mapping given that in many countries, a small proportion of cases are confirmed through laboratory analysis. A similar challenge has been reported by Johansson et al. [67] who point out that clinical records provide a consistent measure of the disease burden in time, and the number of laboratory-confirmed cases would be too low for determination of temporal patterns.

The second limitation was that the model used climatic and environmental factors to predict dengue incidence, but it could not capture potential differences in the distribution of the various serotypes of the dengue virus or host immunity and hence the ADE effect. Serotype-specific immunity, and hence the circulating dengue virus serotypes, is expected to fluctuate in a community overtime, and some of the cyclic epidemics observed might be attributable to these dynamic processes. Primary infection confers life-long protective immunity to an infective serotype, but an introduction of a new serotype 2–3 months later would cause an infection [1]. The third limitation was our inability to include socio-economic variables such as migration patterns, or ability to afford medical care or preventative services, since these actions are expected to influence dengue exposure. These were not available at the spatial scale considered. Areas that require further work include (i) determination of the sensitivity and specificity of the clinical case definition (such as those for), (ii) investigation of the distribution of the DENV serotypes in relation to population immunity, (iii) capacity building on dengue surveillance so that a majority of suspected dengue cases are screened using standard laboratory tests, and (iv) validation of LC through the country.

## Conclusions

The study identified minimum temperature, rainfall, altitude and urban/build-up areas as being significant predictors of dengue incidence in Vietnam. It also revealed that it was important to account for spatial and temporal dependence to account for extra-Poisson distribution of the outcome. Two key products were generated from the study; dengue incidence risk maps showing the distribution of dengue incidence in the wet and dry seasons. The second product was a dengue forecasting model which can generate predictions for forward planning and deployment of interventions.

## Supporting information

S1 Fig Maps prepared from re-analyzed data on altitude (from the Food and Agriculture Organization of the United Nation’s soil data portal) and land cover (downloaded from NASA’s Moderate Resolution Imaging Spectroradiometer [MODIS]) in Vietnam. All these data were used for multivariable modelling but the area under Urban settlement/built-up areas was the only significant variable.
(TIFF)
