# Supplementary figures and images for: Spatiotemporal analysis of historical records (2001–2012) on dengue fever in Vietnam and development of a statistical model for forecasting risk
Source: PLoS One. 2019 Nov 27;14(11):e0224353. doi: 10.1371/journal.pone.0224353 (PMC6881000; doi:10.1371/journal.pone.0224353)

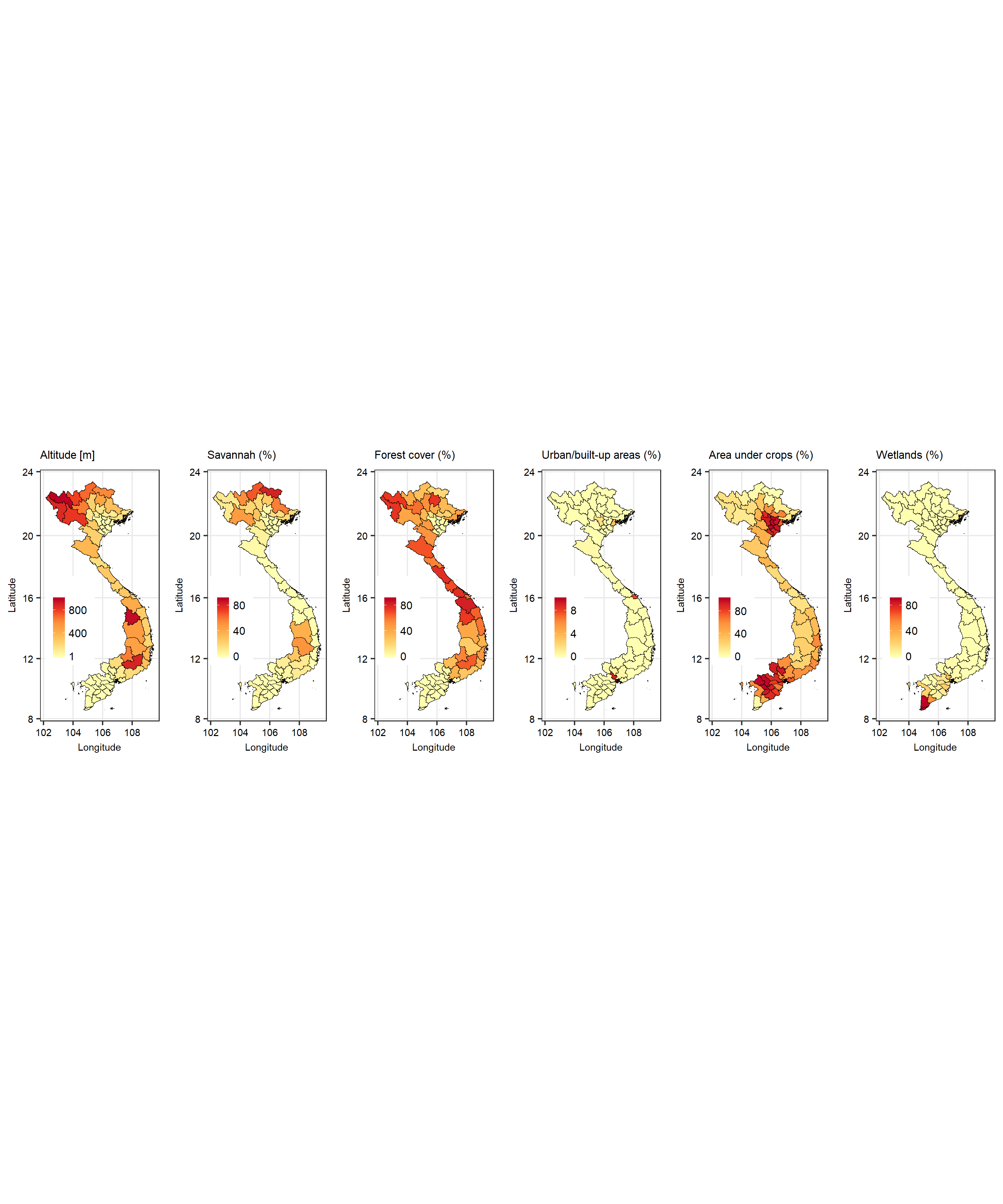

Supplement: S1 Fig — All these data were used for multivariable modelling but the area under Urban settlement/built-up areas was the only significant variable. (TIFF) [file pone.0224353.s001.tiff]
